# Editorial: Insects and changing environments: Emerging perspectives on abiotic stress tolerance mechanisms

**DOI:** 10.3389/fphys.2023.1191318

**Published:** 2023-04-12

**Authors:** Leena Thorat, Jean-Paul Paluzzi, Hans-Joachim Pflüger, Bimalendu B. Nath

**Affiliations:** ^1^ Department of Biology, York University, Toronto, ON, Canada; ^2^ Institute of Biology, Neurobiology, Freie Universität Berlin, Berlin, Germany; ^3^ Stress Biology Research Laboratory, Department of Zoology, Savitribai Phule Pune University, Pune, India; ^4^ MIE-SPPU Institute of Higher Education, Doha, Qatar

**Keywords:** insects, climate change, microplastics, clines, adaptation, physiology, metabolism

Given the enormous and diverse levels of stressors circulating in our environments, living systems, at any given stage of their life cycle are exposed to singular stress factors or a combination of stressors acting on them simultaneously. In this context, stress-homeostatic processes have been central to the field of Stress Response Biology. Among small animals, and as occupants of varied ecological niches, insects exhibit a diverse range of survival tactics. An array of impressive organismal- and cellular-level strategies, and defence machinery enable insect responses against potential stress-induced damages, thereby ensuring their survival and propagation even under challenging conditions.

The field of stress response biology has gained rapid attention in the past few decades, with growing evidence and warnings that our environments are changing. Over the years, insects as representative animal models have served as reliable and tractable model systems to better understand and predict the impacts of changing environments (e.g., Thorat and Nath, 2018). This Research Topic was therefore designed to highlight contributions that investigate meaningful questions relevant to insect abiotic stress tolerance mechanisms and the creative approaches utilized to address those questions.

There were three original research articles and one review published in this issue that cover representative insects from various orders, namely, Coleoptera, Diptera, Hemiptera, Hymenoptera, and Orthoptera. These contributions provide an engaging overview of the adaptation profiles of various insect species and their survival responses to habitat-specific stress conditions. This Research Topic collates recent trends in insect stress biology at the organismal-, behavioural-, cellular, physiological- and molecular-levels using multidisciplinary approaches and cutting-edge tools and techniques.

Khurshid et al. report noteworthy findings on the critical role of antioxidant enzymes and Heat-Shock Protein (HSP) genes in the management of redox homeostasis under heat-inflicted oxidative damage in the green peach aphid, *Myzus persicae*. Cause-effect relations of thermal sensitivity in insects has been an important research area, and this study unravels the implied potential of insect stress biology research in the management of this highly adaptable and polyphagous insect pest that burdens the agricultural industry.

Fudlosid et al. present new insights on the effects of microplastic-based diets administered in the tropical house cricket, *Gryllodes sigillatus*. This study examined whether supplementing a standard diet with either polyethylene microplastic beads (75–105 μm) or polyethylene terephthalate microfibers (<5 mm) would affect selected life history characteristics. Interestingly, while microplastic beads did not have a significant impact on growth and development of *G. sigillatus*, polyethylene fibers were detrimental to the cricket. In summary, the plausible physiological mechanisms for sex-specific impacts of microplastics are yet unclear. However, future investigations examining if and how microplastics are transformed in the insect gut will reinforce our foundational knowledge to devise approaches for tackling the global concerns of plastic pollution.

Menail et al. outline an excellent comparative account of thermal sensitivity and mitochondrial metabolic flexibility in three flying insect species, *Apis mellifera*, *Drosophila melanogaster* and *Leptinotarsa decemlineata*. By measuring oxygen consumption in permeabilized flight muscles from these three species, the authors established how thermal stress is tolerated under conditions involving different oxidative substrates. In conclusion, differential mitochondrial plasticity across insect species demonstrated in this study considerably expands our understanding of the survival dynamics and resilience to thermal stress in some insects over others.

Mayekar et al. offer a comprehensive analysis of the use of clinal variation as a promising tool to monitor population-level correlations between active genomic responses to climate change. Drawing from case studies in *Drosophila*, a cosmopolitan and genetically amenable model, this review highlights interesting broader-scale comparisons of genetic and plastic traits that are not currently well understood. The authors shed light on clinal shift patterns to explain how organisms across latitudes/altitudes are responding to climate change. Taken together, this review proposes the role of clinal variation studies to complement measures of mitigating climate change.

## Conclusion

Overall, this Research Topic provides original research data and an in-depth review that enlighten us on comparative stress-induced adaptive frameworks in various insect species. Additionally, we anticipate that findings from these studies will pave the way for translational research as well as help leverage the implied potential of the field of stress response biology in supporting evidence-based policymaking in climate change management and mitigation, and biodiversity conservation.

As co-editors, we enjoyed working with the authors and we sincerely thank them for submitting their interesting work to this topic Research Topic.

With more exciting discoveries awaited for the future, we envisage that this Research Topic will serve as a foundation to stimulate further interest in gaining cross-axes insights from evolutionary, cellular, eco-physiological and biochemical perspectives on insects.

We hope our readers will enjoy this work, as much as we enjoyed putting it together for you!

## Dedication statement

This Research Topic is dedicated to the memory of Prof. Hans-Joachim Pflüger who passed away on 25 January 2022.

Prof. Pflüger, whom we dearly called ‘Jochen’ was a Professor of Functional Neuroanatomy/Neurobiology at Freie Universität Berlin, Germany. As one of the founding members and one of the presidents of the German Neuroscience Society, Jochen was a backbone to the Neurosciences community in Germany and Europe.

Jochen’s research focused on how sensory feedback mechanisms, neuronal networks, and neuromodulation lead to meaningful behavior in insects and other arthropods. When not in the lab, Jochen was a keen birder, loved nature and enjoyed taking students to field excursions.

Apart from his professional credentials, Jochen was a wonderful human being who touched the lives of many! As his co-editors, we thank Jochen for his unwavering support, generous scientific and technical advice and for co-editing the submissions published in this Research Topic Research Topic.
